# Light Promotes the Immobilization of U(VI) by Ferrihydrite

**DOI:** 10.3390/molecules27061859

**Published:** 2022-03-13

**Authors:** Yun Wang, Jingjing Wang, Zhe Ding, Wei Wang, Jiayu Song, Ping Li, Jianjun Liang, Qiaohui Fan

**Affiliations:** 1 Northwest Institute of Eco-Environment and Resources, Chinese Academy of Sciences, Lanzhou 730000, China; 13080353116@163.com (Y.W.); jjwang2017@lzu.edu.cn (J.W.); dingzhe18@mails.ucas.ac.cn (Z.D.); ucasww@163.com (W.W.); sjy0784@163.com (J.S.); liangjj@lzb.ac.cn (J.L.); fanqh@lzb.ac.cn (Q.F.); 2 Key Laboratory of Strategic Mineral Resources of the Upper Yellow River, Ministry of Natural Resources, Lanzhou 730000, China; 3 Key Laboratory of Petroleum Resources, Lanzhou 730000, China

**Keywords:** ferrihydrite, photocatalysis, U(VI/IV), uranium peroxides, immobilization

## Abstract

The environmental behaviors of uranium closely depend on its interaction with natural minerals. Ferrihydrite widely distributed in nature is considered as one main natural media that is able to change the geochemical behaviors of various elements. However, the semiconductor properties of ferrihydrite and its impacts on the environmental fate of elements are sometimes ignored. The present study systematically clarified the photocatalysis of U(VI) on ferrihydrite under anaerobic and aerobic conditions, respectively. Ferrihydrite showed excellent photoelectric response. Under anaerobic conditions, U(VI) was converted to U(IV) by light-irradiated ferrihydrite, in the form of UO_2+x_ (x < 0.25), where •O_2_^−^ was the dominant reactive reductive species. At pH 5.0, ~50% of U(VI) was removed after light irradiation for 2 h, while 100% U(VI) was eliminated at pH 6.0. The presence of methanol accelerated the reduction of U(VI). Under aerobic conditions, the light illumination on ferrihydrite also led to an obvious but slower removal of U(VI). The removal of U(VI) increased from ~25% to 70% as the pH increased from 5.0 to 6.0. The generation of H_2_O_2_ under aerobic conditions led to the formation of UO_4_•xH_2_O precipitates on ferrihydrite. Therefore, it is proved that light irradiation on ferrihydrite significantly changed the species of U(VI) and promoted the removal of uranium both under anaerobic and aerobic conditions.

## 1. Introduction

The rapid development of nuclear power requires an increasing demand for uranium, the primary fuel for nuclear reactors [1,2]. In recent years, the worldwide natural uranium production is between 55,000 and 65,000 tons per year [3]. However, the nuclear fuel cycle including mining, milling, and power production typically generates a substantial quantity of uranium waste. Due to the serious health hazards caused by uranium [4], investigation on the environmental fate of uranium is extremely important. Under natural conditions, most uranium exists as hexavalent uranyl (U(VI)) with high solubility and mobility [5]. Once released to the environment, U(VI) would be adsorbed by natural media or reduced to immobile U(IV) under reduction conditions [5,6,7,8,9], subsequently affecting the transportation and environmental risks of uranium. Therefore, the interactions between U(VI) and natural media such as minerals have received great attentions.

Iron is one of the most important elements on earth, and iron (oxyhydr)oxides are widely distributed in natural environments [10,11]. Ferrihydrite, a poorly ordered iron oxide, can be found in soils, sediments, rocks, and waters [11,12]. Because of the relatively large surface area and abundant reactive sites, ferrihydrite is generally regarded as an outstanding scavenger for cations and anions [13,14,15,16]. Especially, ferrihydrite has been verified to be an excellent scavenger for arsenate [16]. It has been proven that ferrihydrite also shows excellent adsorption affinity for U(VI), which was much higher than goethite and magnetite [8]. Owing to the wide distribution of ferrihydrite in nature, the environmental fate of uranium should be markedly impacted by ferrihydrite. Although iron oxides have received much attention owing to their excellent adsorption ability, their photocatalytic properties are sometimes ignored. It is well known that the light irradiation on semiconductor minerals would excite electrons (*e*^−^) from the valence band (VB) to the conduction band (CB) and result in the charge separation [17,18]. The photogenerated electrons, holes, together with the subsequently produced ^1^O_2_, •O_2_^−^, •OH, and H_2_O_2_ can induce complex redox reactions. which would further impact the environmental fate of various metal ions and organic matters [18,19,20,21]. Studies have indicated that some iron oxides can act as natural photocatalysts to catalyze the oxidation of organic matters [22,23,24], and ferrihydrite could also act as a semiconductor material for Mn(II) oxidation [25,26] and organic matter degradation [23]. Hence, ferrihydrite is expected to show photocatalytic properties and induce redox reactions. For U(VI), it has been suggested that the presence of semiconductors would lead to rapid reduction of U(VI) under anaerobic conditions, forming reduced deposits on solid surface [27,28,29,30,31,32,33,34]. Therefore, it is considered that light-irradiated ferrihydrite would bring change to the chemical state of uranium and subsequently impact its environmental behaviors, deserving further exploration.

The objective of this research is to probe the influence of the photocatalytic reactions induced by ferrihydrite on the environmental behaviors of U(VI). Uranium exists in both anaerobic and aerobic environments, and oxygen generally brings significant influence on the photo-generated species. Therefore, the reactions were investigated both in N_2_ and air atmosphere to simulate the anaerobic and aerobic conditions in nature. The findings may help improve understanding the geochemical behaviors of uranium in nature.

## 2. Results and Discussion

### 2.1. Physicochemical Properties of Ferrihydrite

**Figure 1:**
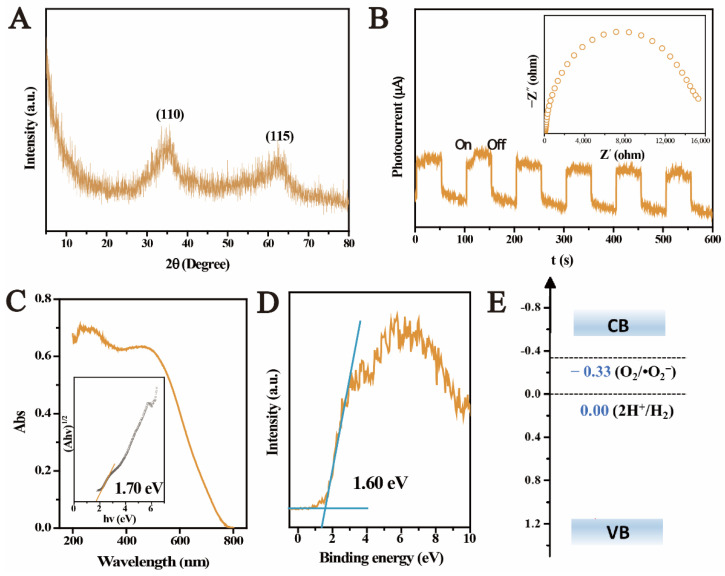
Physicochemical properties of ferrihydrite, XRD pattern (**A**), time-dependent photocurrent measurement and EIS (inset) (**B**), UV–Vis diffuse reflectance spectra (**C**) and the optical band gap energy (inset), valence band X-ray photoelectron spectroscopy (VB-XPS) (**D**), and the band edge positions (**E**).

Figure 1A depicts the XRD pattern of the as-prepared ferrihydrite. Two peaks centered at about 34.8° and 62.2° corresponded to the (110) and (115) planes of 2-lines ferrihydrite [35]. No signal from impurities was found in the XRD pattern, suggesting that the prepared sample was pure ferrihydrite. The photoelectrochemical (PEC) properties of ferrihydrite were further tested. As shown in Figure 1B, the ferrihydrite electrode showed an immediately enhanced photocurrent upon light illumination, indicating that ferrihydrite had a sensitive light response and efficient photo-charge separation ability. The electrochemical impedance spectroscopy (EIS) (inset in Figure 1B) also supported this. A small arc radius on the EIS Nyquist plot was observed, corresponding to a fast interfacial charge transfer in ferrihydrite. Moreover, the arc radius became smaller under light irradiation (Figure S1 in the Supporting Information), which was mainly due to the difference in the electron density of the electrode. The decreased resistance value suggested enhanced electron transport performance under light illumination [36]. DRS was used to investigate the optical absorbance of ferrihydrite. From Figure 1C, ferrihydrite exhibited an obvious absorbance for visible light. The corresponding bandgap (Eg) of ferrihydrite was estimated to be 1.70 eV using the Tauc plots transferred from Kubelka–Munk function (inset in Figure 1C) [37]. The VB-XPS was collected to evaluate the valance band potential (E_VB_) of ferrihydrite. From Figure 1D, the VB maximum (E_VBM_) of ferrihydrite located at 1.60 eV. The E_VB_ was determined to be 1.16 V (vs. NHE) according to the Eqn. [38]:

E_NHE_ = Φ + E_VBM_ − 4.44 (1)

where E_NHE_ was standard electrode potential, and Φ represents the electron work function of the analyzer (4.00 eV). Combining the above results, the conduction band potential (E_CB_) of ferrihydrite was −0.54 V vs. NHE (Figure 1E). Therefore, it was proved that prepared 2-lines ferrihydrite showed excellent optical absorbance, sensitive photoelectric response, and moderate band structures that is promising for the photocatalysis for U(VI) [27]. In addition, the EIS and Fe 2p spectra of ferrihydrite remained unchanged after the photocatalytic reactions (Figures S2 and S3), indicating the stability of ferrihydrite under light.

### 2.2. The Photocatalytic U(VI) Reduction under Anaerobic Conditions

**Figure 2:**
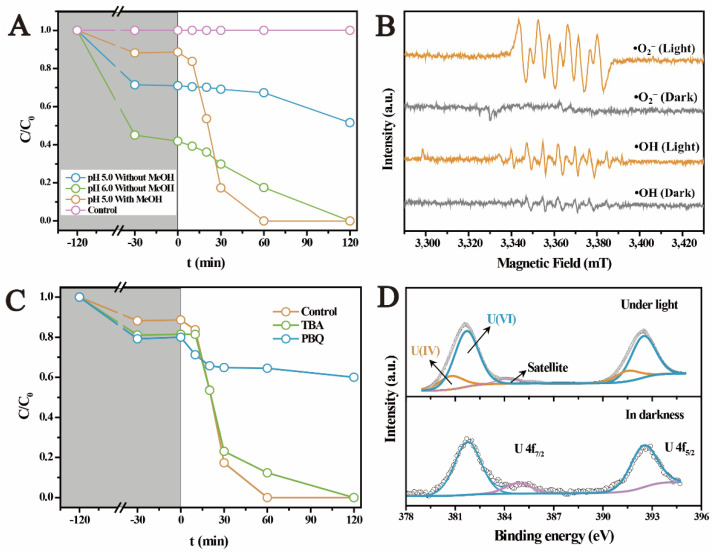
The photocatalytic reduction of U(VI) by ferrihydrite under anaerobic conditions (**A**), ESR spectra of •OH and •O_2_^−^ radicals (**B**), the photocatalytic kinetics of U(VI) on ferrihydrite by the addition of PBQ (scavenging•O_2_^−^) and TPA (scavenging •OH) (**C**), and U 4f XPS spectra for the products after adsorption and photocatalytic reaction (**D**).

Figure 2A displays the U(VI) removal on ferrihydrite in N_2_ atmosphere. After interaction in darkness for 2 h, adsorption equilibrium was achieved and removed ~30% of U(VI) at pH 5.0, while it increased to ~50% of U(VI) at pH 6.0. However, under light, the amount of solution U(VI) was gradually reduced at pH 5.0, and approximately 20% of U(VI) was removed after light irradiation for 120 min. U(VI) removal occurred much faster at pH 6.0, where all U(VI) was eliminated during the same period. By contrast, the blank test without ferrihydrite as catalyst showed that U(VI) was hardly removed under light (Figure 2A), indicating the negligible self-photolysis of U(VI). Therefore, it was evidenced that light-irradiated ferrihydrite promoted the U(VI) removal under anaerobic conditions, which should be closely related to the semiconductor properties of ferrihydrite. Recent studies proved that U(VI) was rapidly reduced by light-irradiated semiconductors, especially under anaerobic conditions [28,29,30,31,32,33,34]. The enhanced photocatalytic U(VI) reduction at higher pH was attributed to the weaker competition of H^+^ for the photogenerated reactive reductive species [34]. Moreover, in the presence of methanol, despite the slightly inhibited adsorption of U(VI), the photocatalytic U(VI) reduction was obviously promoted (within 60 min) (Figure 2A). This suggests that the presence of such kind of low molecular weight organic matters in nature may promote the reduction of U(VI). Typically, these low molecular weight organic matters including methanol, formate, ascorbic acid, and ethanol could act as the scavengers for the photogenerated holes, prolonging the lifetime of electrons [39]. In addition, some reductive free radicals (such as •CO_2_^−^) generated during the oxidation of these organic matters could also contribute to the U(VI) reduction [40].

To clarify the mechanisms for the photocatalytic U(VI) reduction on ferrihydrite, the reactive reductive species were investigated using ESR and free radical capture experiment. Figure 2B shows that no signal was found in the ESR spectra in darkness, whilst obvious characteristics of DMPO-•O_2_^−^ and DMPO-•OH were detected upon illumination. This confirmed the generation of •O_2_^−^ and •OH radicals on light irradiated ferrihydrite. The roles of •O_2_^−^ and •OH in reducing U(VI) were determined with radical capture experiments, as exhibited in Figure 2C. Obviously, the photocatalytic reduction of U(VI) was remarkably inhibited by p-benzoquinone (PBQ), while tertiary butanol (TBA) slightly affected the reduction of U(VI). Therefore, •O_2_^−^ radicals were proved to be the dominant reductive species for U(VI) reduction on ferrihydrite [33,40].

**Figure 3:**
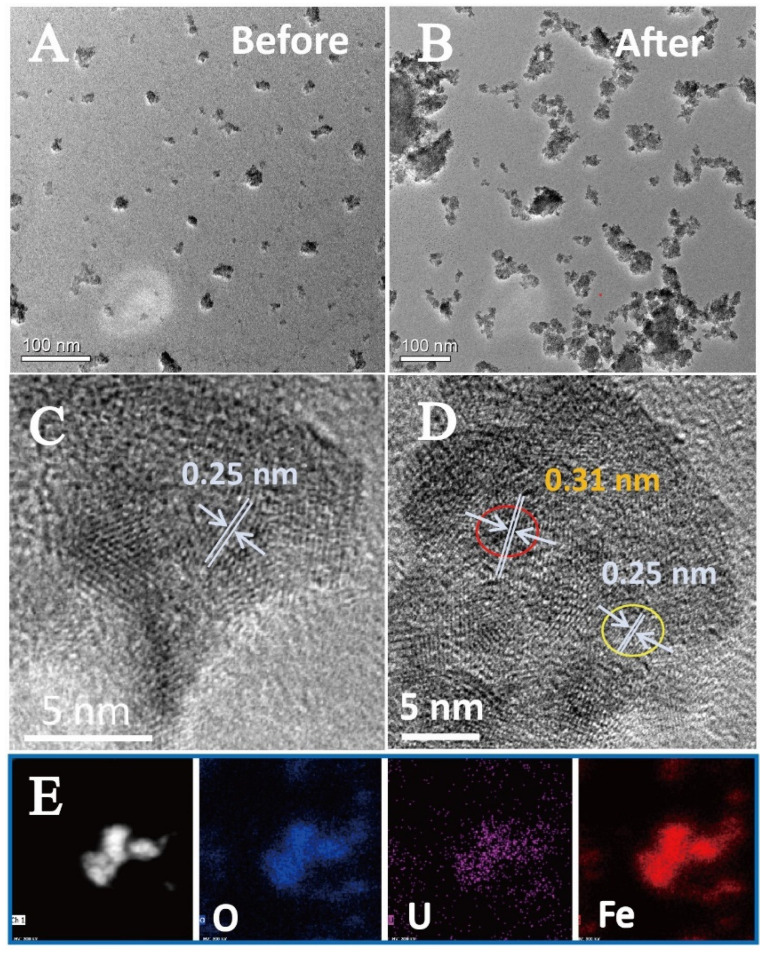
TEM images of ferrihydrite before (**A**,**C**) and after the photocatalytic reactions (**B**,**D**), and the element mapping of uranium loaded ferrihydrite (**E**).

The transformation of U(VI) on ferrihydrite during the photocatalytic process was further determined by XPS investigation. Figure 2D compares the U 4f spectra of the uranium loaded ferrihydrite before and after light irradiation. In darkness, two peaks appeared at 381.9 eV and 392.9 eV, corresponding to the characteristics of U(VI) adsorbed on ferrihydrite [34]. After irradiating the sample by light, U 4f_5/2_ and U 4f_7/2_ peaks shifted by ~0.4 eV to lower binding energy, both of which could be deconvoluted into two peaks, respectively. Comparing with the results of U(VI) adsorbed on ferrihydrite, new peaks appeared at 380.8 eV and 391.6 eV, corresponding to the characteristics of U(IV) [34]. This result confirmed the reduction of U(VI) on light-irradiated ferrihydrite. However, XPS analysis also indicated that uranium partially existed in its hexa-valence in the products after the photocatalytic reactions. This phenomenon was similar to all studies involving the photocatalytic U(VI) reduction [27,28,29,30,31,32,33,34]. It was considered that the products were UO_2+x_ (x < 0.25) [40,41,42], or the reduced products was re-oxidized again on the surface [34]. Figure 3A shows the morphology of raw ferrihydrite. Pristine ferrihydrite formed relatively uniform aggregates with diameter of about 20 nm. Comparing with the small aggregates of raw ferrihydrite, the size of aggregates became much bigger after the photocatalytic reactions (Figure 3B). The HRTEM image of raw ferrihydrite showed that the space of the lattice fringe was about 0.25 nm (Figure 3C), corresponding to the (110) plane of ferrihydrite [43]. Upon light illumination, additional fringes with a spacing of 0.31 nm were observed (Figure 3D), which is in accord with the characteristic of the (111) facet of UO_2+x_ [33,40]. The formation of the uranium-containing deposits on ferrihydrite surface could be further verified by the elemental mapping (Figure 3E), where uranium uniformly deposited on ferrihydrite surface. The above results proved that U(VI) was photocatalytically reduced by ferrihydrite, forming uranium oxides with crystal structures similar to UO_2_. In summary, it was proved that light irradiated ferrihydrite could induce photocatalytic reactions under anaerobic conditions, reducing U(VI) to insoluble U(IV) deposits, which subsequently changed the behaviors and fate of uranium in nature. This may help to better understand the environmental behaviors of uranium in anerobic environment, such as subsurface waters containing semiconductor minerals.

### 2.3. The Photocatalytic Immobilization of U(VI) under Aerobic Conditions

**Figure 4:**
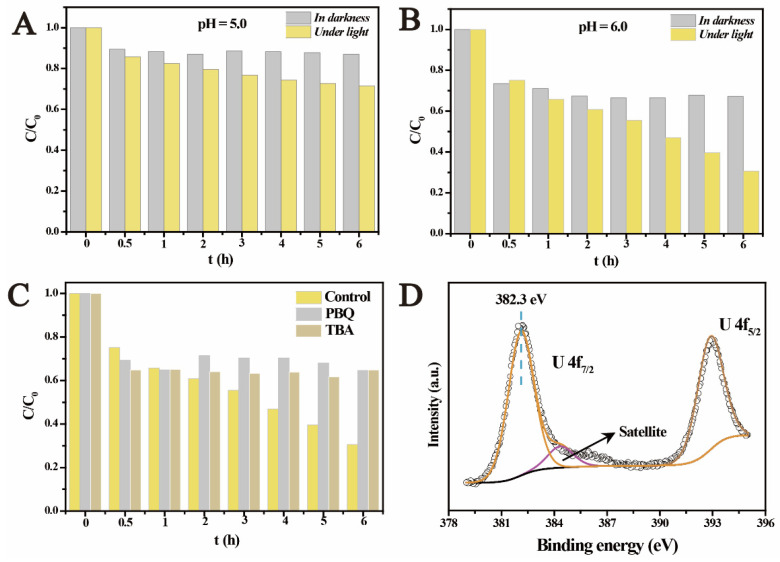
The photocatalysis of U(VI) by ferrihydrite in open air at pH 5.0 (**A**) and 6.0 (**B**), free radicals capture experiments under aerobic conditions (**C**), and U 4f spectrum of the obtained products for the photocatalysis of U(VI) in air (**D**).

Comparing with the reactions under anaerobic conditions, it is more valuable to explore the photocatalysis under aerobic conditions. This is mainly because more natural semiconductors are generally exposed to both light irradiation and the atmosphere. However, it has been proved by numerous studies that the photocatalytic U(VI) reduction mostly occurred in anaerobic environment [27,28,29,30,31,32,40]. This was mainly because: (1) the exposure of the catalytic system to air would increase the concentration of dissolved oxygen, which furthered competition for the photogenerated electrons [44]; (2) the presence of excess oxygen molecules would result in more oxidative species such as •OH, which strongly suppressed the reduction of U(VI) [40]; and (3) high concentration of dissolved oxygen easily made the reduced products re-oxidized [34]. In fact, the limited photocatalytic U(VI) reduction under aerobic conditions has become one major problem limiting its application [27]. Recently, few studies have achieved the photocatalytic U(VI) reduction in atmosphere by designing catalysts with proper band structures to reduce reactive oxidative species (ROS) [33]. However, the band structures of the natural semiconductors are fixed. Therefore, it is critical to evaluate if the behaviors of uranium can be affected by the photocatalytic reactions under aerobic conditions. Figure 4A,B show the photocatalysis of U(VI) over ferrihydrite in air atmosphere at different pHs. In darkness, the adsorption equilibrium was reached within 0.5 h, where 10% and 25% of U(VI) was adsorbed at pH 5.0 and 6.0, respectively. Compared with the results obtained in anaerobic environment, U(VI) adsorption in air atmosphere was weaker. This was mainly because the continuous bubbling of air into the suspension increased the concentration of dissolved carbonates, which would result in the inhabitation on U(VI) adsorption [42]. Upon light irradiation, unexpectedly, U(VI) was gradually removed from solution. ~25% of U(VI) was removed at pH 5.0 after light irradiation for 6 h, while the removal of U(VI) reached ~70% at pH 6.0. Compared with the photocatalytic U(VI) reduction in anaerobic environment, the photocatalysis induced U(VI) removal in air atmosphere was slower but still effective.

**Figure 5:**
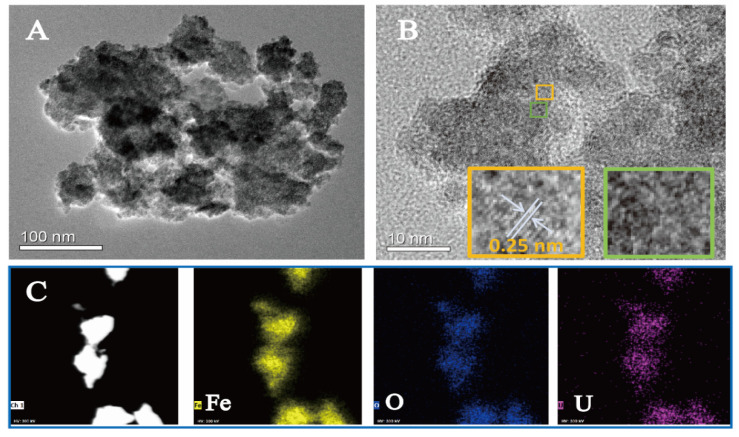
TEM images of ferrihydrite after the photocatalysis of U(VI) in air (**A**,**B**), and the corresponding element distribution (**C**).

To clarify this unexpected result, free radicals capture experiments were conducted. Similar to the reactions under the anaerobic conditions, the presence of PBQ inhibited U(VI) removal. Surprisingly, the removal of U(VI) was completely restrained in the presence of TBA, which acted as the scavenger for •OH (Figure 4C). This suggested a new reaction process for U(VI) removal under aerobic conditions, which was different from that under anaerobic conditions. For in-depth understanding the reaction mechanisms, the products were analyzed by XPS investigation. It is interesting to notice that the U 4f signals of the products appeared at 382.3 eV and 393.1 eV, respectively (Figure 4D), being higher than the characteristics of adsorbed U(VI) and U(IV). This indicates that the removal of U(VI) by light irradiated ferrihydrite in air atmosphere was not caused by the reduction of U(VI) or simple adsorption, being different from the results under the anaerobic conditions. TEM images show that the size of the observed ferrihydrite particles also increased obviously after the photocatalytic reactions (Figure 5A). The deposit of uranium-containing products was confirmed by the element distribution, as shown in Figure 5C. However, from the HRTEM images (Figure 5B), the products only clearly show the lattice fringes of ferrihydrite and the other part showed no fringe, indicating the formation of uranium products with non-crystal structure. This proved that the products of the photocatalysis of U(VI) under aerobic conditions differed from that obtained in N_2_ atmosphere, and should be caused by different mechanisms. It is well known that H_2_O_2_ is a common product of the photocatalytic reactions in air atmosphere, and H_2_O_2_ was usually regarded as one main ROS [45]. It was considered that the photogenerated H_2_O_2_ would react with uranyl to form UO_4_•xH_2_O (s) precipitates. The reaction occurred as follows [46,47]:

UO_2_^2+^ + H_2_O_2_ + xH_2_O = UO_4_•xH_2_O (s) + 2H^+^ (2)

**Figure 6:**
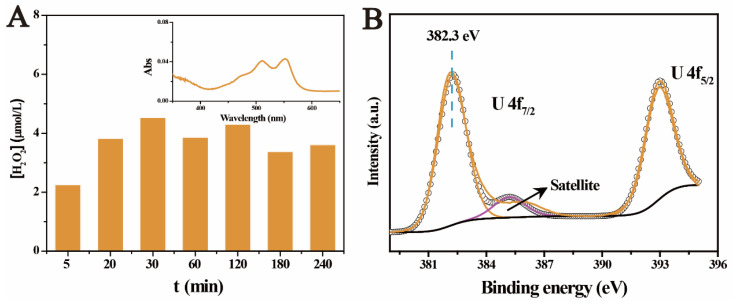
Generation of H_2_O_2_ after light irradiation on ferrihydrite (**A**), and the U 4f spectrum of UO_4_•xH_2_O (**B**).

Actually, this has become one of the traditional processes for producing the so-called ‘yellowcake’. To verify this, the generation of H_2_O_2_ on ferrihydrite was detected by adding N,N-diethyl-p-phenylenediamine (DPD) to the sample after light irradiation [48]. As shown in Figure 6A, the generation of H_2_O_2_ was verified by the characteristic red color of DPD•^+^ and strong absorbance at ~550 nm in the UV–vis absorbance spectrum (inset in Figure 6A). The concentration of H_2_O_2_ remained at about 3.5 × 10^−6^ mol/L. The critical role of H_2_O_2_ in the photocatalytic removal of U(VI) under aerobic conditions was also supported by the results of the radical capture experiments. The suppressing of •O_2_^−^ and •OH radicals restrained the generation of H_2_O_2_, and subsequently made the removal of U(VI) hardly occurred. Furthermore, the XPS spectra of UO_4_•xH_2_O standard was obtained and shown in Figure 6B. The binding energy of UO_4_•xH_2_O standard located at 382.3 eV (U 4f_7/2_) and 391.1 eV (U 4f_5/2_), respectively, which were the same with the photocatalysis products of U(VI) obtained in air atmosphere. This also supported the formation of UO_4_•xH_2_O deposits on ferrihydrite under aerobic conditions. In general, it was proved that the irradiation of light on ferrihydrite under the aerobic conditions led to the generation of H_2_O_2_, which subsequently resulted in the precipitation of uranyl, in the form of UO_4_•xH_2_O (s).

## 3. Materials and Methods

### 3.1. Materials

UO_2_(NO_3_)_2_•6H_2_O was dissolved in Milli-Q water to prepare U(VI) solution. Ferrihydrite was prepared using the method reported by Wang et al. [49]. Briefly, 1.0 mol/L NaOH solution was added into 500 mL 20 mmol/L Fe^3+^ solution until the pH reached 7.5. After stirring for 2 h, the brown precipitates were filtered and the solid was washed for several times by pure water. Finally. the obtained products were freeze dried and ground to pass a 200-mesh screen. UO_4_•xH_2_O standard was prepared by adding H_2_O_2_ into uranyl solution. The obtained yellow precipitates were then separated and freeze dried. The X-ray diffraction (XRD) pattern of the products was shown in Figure S4.

### 3.2. Photocatalysis Test

A total of 9.0 mg of ferrihydrite was mixed with 15 mL 0.1 mmol/L U(VI) solution. The pH was adjusted by using HCl and NaOH solution. For the experiments under anaerobic conditions, the reaction cell was bubbled with N_2_ for 2 h in the dark to remove O_2_. As for the experiments under aerobic conditions, the reaction cell was bubbled with air. To simulate the irradiation of sunlight, a 300 W Xe lamp was used to illuminate the suspension, while control experiments were conducted in darkness. At desired time, 1.0 mL of the suspension was taken out and filtered to obtain the supernatant, and the residual U(VI) in solution was measured using the method in reference [34].

### 3.3. Characterization

The samples were characterized by transmission electron microscopy (TEM, Fei Tecnai G2 F30), X-ray diffraction (XRD, D/Max-2400, Rigaku, Tokyo, Japan), and UV–vis diffuse reflection spectrum (DRS, UV-2550, Shimadzu, Kyoto, Japan). The chemical states of uranium and valence band of the catalysts were obtained by X-ray photoelectron spectroscopy (XPS, Thermo Scientific ESCALAB Xi^+^, Waltham, MA, USA) with Al-Kα X-ray source. The binding energies were calibrated by C 1 s at 284.8 eV. The electron spin resonance (ESR) was gained on Bruker A300 spectrometer, and 5,5-dimethyl-l-pyrroline N-oxide (DMPO) was used as the probe. The electrochemical measurements were conducted on electrochemical workstation (CHI-600e, Chenghua, Shanghai, China). The concentration of H_2_O_2_ was measured using the method in reported research [48].

## 4. Conclusions

In summary, we investigated the photocatalysis of U(VI) over ferrihydrite under anaerobic and aerobic conditions, respectively. Ferrihydrite exhibited excellent photoelectric properties. Under anaerobic conditions, U(VI) was photocatalytically reduced by light-irradiated ferrihydrite, which acted as a natural semiconductor. U(VI) was reduced to U(IV) by •O_2_^−^ radicals, in the form of UO_2+x_ (x < 0.25) precipitates. On the contrary, a considerable amount of H_2_O_2_ was generated by irradiating ferrihydrite in open air. The presence of H_2_O_2_ led to the precipitation of uranyl, forming UO_4_•xH_2_O (s). Therefore, it was proved that light irradiation on ferrihydrite would change the species of uranium both under anaerobic and aerobic conditions, making soluble U(VI) to convert to its immobile forms and promoted U(VI) removal. This would subsequently result in the change of the environmental fate of uranium in nature. These results should be helpful for better understanding and evaluating the behaviors of uranium in the natural environment. Moreover, it may also reveal the important roles of semiconductor minerals in tuning the geochemical behaviors of elements. Further clarification of the photocatalytic activities of other natural semiconductor minerals and the impacts of environmental factors on the catalytic reactions would enhance its scalability.

## Data Availability

Data are contained within the article.

## Supplementary Materials

The following supporting information can be downloaded at: https://www.mdpi.com/article/10.3390/molecules27061859/s1. Figure S1: Comparison of EIS measurements under illumination condition with that under dark condition; Figure S2: EIS of ferrihydrite before and after the photocatalytic reactions under anaerobic and aerobic conditions; Figure S3: Fe 2p spectra of ferrihydrite before and after the photocatalytic reactions under anaerobic and aerobic conditions; Figure S4: The XRD pattern of the reaction products of uranyl with H_2_O_2_.

## Sample Availability

Samples of the compounds are available from the authors.
