# Supplementary material for: Light Promotes the Immobilization of U(VI) by Ferrihydrite
Source: Molecules. 2022 Mar 13;27(6):1859. doi: 10.3390/molecules27061859 (PMC8950992; doi:10.3390/molecules27061859)
Supplement: Supplementary file 1 [file molecules-27-01859-s001.zip › molecules-1579542-supplementary.pdf]

Supporting Information

Light promotes the immobilization of U(VI) by ferrihydrite

Yun Wang[1,2,3], Jingjing Wang[1,3], Zhe Ding[1,2,3], Wei Wang[1,2], Jiayu Song[1,2], Ping Li[1,2,3,*], Jianjun Liang[1,2,3,], Qiaohui Fan[1,2,3,]

[1] Northwest Institute of Eco-Environment and Resources, Chinese Academy of Sciences, Lanzhou 730000, China;
[2] Key Laboratory of Strategic Mineral Resources of the Upper Yellow River, Ministry of Natural Resources, Lanzhou 730000, China
[3] Key Laboratory of Petroleum Resources, Lanzhou 730000, China
* Correspondence: liping@lzb.ac.cn

Contents: Figures S1–S4

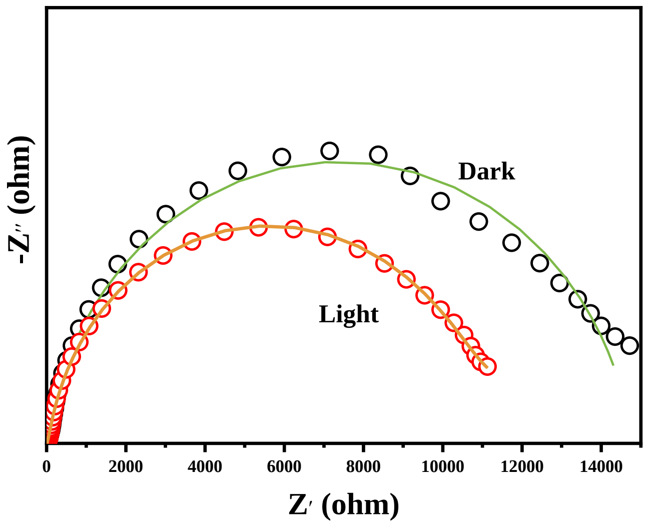

Figure S1. Comparison of EIS measurements under illumination condition with that under dark condition. The calculated R for the catalyst under light and in darkness were 7388 Ohm and 9020 Ohm, respectively.

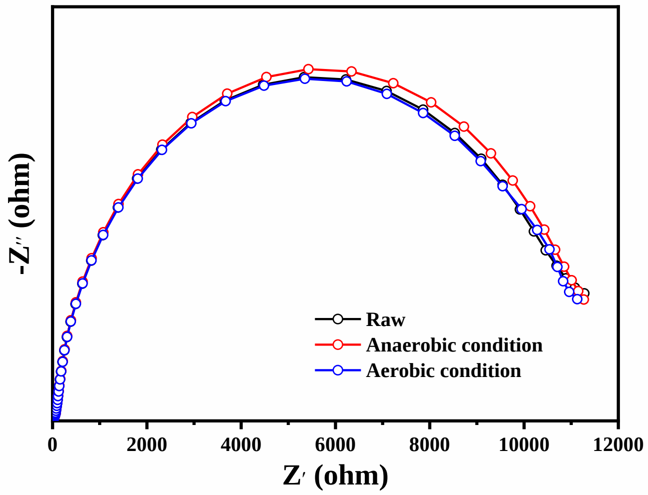

Figure S2. EIS of ferrihydrite before and after the photocatalytic reactions under anaerobic and aerobic conditions.

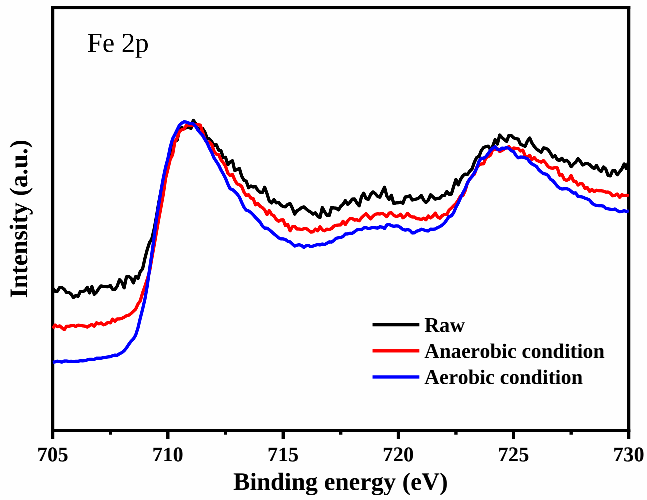

Figure S3. Fe 2p spectra of ferrihydrite before and after the photocatalytic reactions under anaerobic and aerobic conditions.

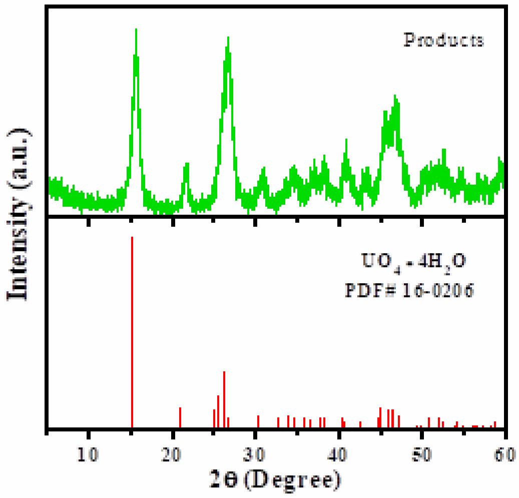

Figure S4. The XRD pattern of the reaction products of uranyl with H_2O_2.
